# Synthesis and Structural Modulation of Nanoporous Copper Films by Magnetron Sputtering and One-Step Dealloying

**DOI:** 10.3390/ma17235705

**Published:** 2024-11-21

**Authors:** Jinglei Li, Bin Yu, Yunfei Ran, Yalong Liu, Xiangyu Fei, Jiameng Sun, Fuquan Tan, Guanhua Cheng, Ying Zhang, Jingyu Qin, Zhonghua Zhang

**Affiliations:** Key Laboratory for Liquid-Solid Structural Evolution and Processing of Materials (Ministry of Education), School of Materials Science and Engineering, Shandong University, Jingshi Road 17923, Jinan 250061, China; li15634100279@163.com (J.L.); yubin0225@163.com (B.Y.); 17686689897@163.com (Y.R.); 17862712217@163.com (Y.L.); xy_fey@yeah.net (X.F.); sunxxxxjm@163.com (J.S.); z18779080029@163.com (F.T.); guanhua.cheng@sdu.edu.cn (G.C.); qinjy@sdu.edu.cn (J.Q.)

**Keywords:** nanoporous copper, dealloying, magnetron sputtering, structural regulation

## Abstract

Nanoporous copper (np-Cu) has attracted much more attention due to its lower cost compared to other noble metals and high functionality in practical use. Herein, Al_100−x_Cu_x_(x = 13–88 at.%) precursor films with thicknesses of 0.16–1.1 μm were fabricated by varying magnetron co-sputtering parameters. Subsequently, utilizing a one-step dealloying strategy, a series of np-Cu films with ligament sizes ranging from 11.4–19.0 nm were synthesized. The effects of precursor composition and substrate temperature on the microstructure of np-Cu films were investigated. As the atomic ratio of Cu increases from 15 to 34, the np-Cu film detached from the substrate gradually transforms into a bi-continuous ligament-channel structure that is well bonded to the substrate. Furthermore, the novel bi-layer hierarchical np-Cu films were successfully prepared based on single-layer nanoporous films. Our findings not only contribute to the systematic understanding of the modification of the morphology and structure of np-Cu films but also offer a valuable framework for the design and fabrication of other non-noble nanoporous metals with tailored properties.

## 1. Introduction

In recent years, nanoporous metals have been extensively applied in catalysis [1,2], battery electrodes [3,4], energy storage [5,6,7], and biomedical fields [8,9] due to their high porosity and specific surface area [10]. Dealloying [11], which involves selective dissolution of reactive elements and diffusion/spontaneous organization of less reactive elements, emerges as a straightforward and efficient method to fabricate nanoporous metals [12,13]. Among various metals, nanoporous copper (np-Cu) has received much attention owing to its low cost and high catalytic performance comparable to noble metals [14,15]. Bai et al. [16] reported an np-Cu material featuring a multilevel pore structure, exhibiting excellent performance in solar water evaporation comparable to nanoporous Au and Ag films. Regulating the microstructure of nanoporous metals, including np-Cu, is urgently needed for their properties and applications. For example, Hyun et al. [17] prepared a 3D hierarchically porous Au comprising interconnected macro/nano porous channels. Such a multiscale porous structure can not only provide microchannels for facilitating the rapid transport of electrolyte ions but also maintain a high specific surface area for increasing active sites [18]. Consequently, the 3D hierarchically porous Au exhibited a highly efficient mass activity, which was 3.96 times higher than that of conventional dealloyed nanoporous Au. At present, various strategies based on precursor design (composition, phase constituent, and distribution) [19], the control of the dealloying process [20,21] and post-dealloying treatment [22] are mainly utilized to modulate the structure and composition of nanoporous metals. Notably, considering the direct correlation between morphology features of precursor alloy and nanoporous metals, a large number of studies have focused on the precursor design. Wang et al. [23] proposed that nanoporous Ag ribbons with diverse morphologies can be obtained by adjusting the composition of Al-Ag precursor alloys, confirming that the ligament size of the nanoporous Ag increases with increasing Ag contents of precursor alloys. In addition, Chauvin et al. [24] fabricated a series of Al-Ag alloy films by co-sputtering and further explored the relation between initial morphology and composition of alloy films and the final nanoporous structure after dealloying.

Up to now, the most commonly used methods for synthesizing precursor alloys include melt-solidification [25], arc melting [26], and melt-spinning [27]. Compared with the above methods, magnetron sputtering, as a physical vapor deposition method, has great advantages in preparing precursors for nanoporous materials. On the one hand, the thickness and size of precursor films can be precisely tailored by adjusting sputtering parameters [19,24]. Even thickness below 10 nm can be achieved. On the other hand, bi-layer or multi-layer precursor films can be fabricated by controlling constituent distribution during sputtering, subsequently forming a composite functional material for further application.

In this work, magnetron co-sputtering was applied to precisely control Al-Cu alloy films with different compositions. Subsequently, a one-step dealloying strategy was adopted to obtain a series of np-Cu films with various morphologies. The effect of sputtering parameters (co-sputtering power, time, and various substrates) on the composition, thickness, and morphology of Al-Cu alloy films was systematically studied. The influence of precursor alloy composition/morphology and substrate temperature on the np-Cu films was further explored. Eventually, on the basis of preparing single-layer np-Cu films, bi-layer precursor films were deposited to fabricate bi-layer np-Cu films with different hierarchical structures. This work achieved the regulation of microstructure in nanoporous Cu by designing precursors and adjusting substrate temperature. More importantly, the preparation of bi-layer nanoporous Cu films with hierarchical structures can bring inspirations for exploring new functional nanoporous materials.

## 2. Experimental

### 2.1. Material Preparation

The direct current (DC) and radio frequency (RF) magnetron co-sputtering techniques were utilized to fabricate Al-Cu thin films using metallic Al and Cu targets (diameter: 60 mm; thickness: 4 mm; purity: 99.99 wt.%) (Dream Material Technology Co., Ltd., Beijing, China). Before co-sputtering, electrodeposited Cu foils (ED Cu foils; thickness: 9 ± 1 μm) and rolled Cu foils (RA Cu foils; thickness: 30 ± 2 μm) (Canrd Technology Co., Ltd., Dongguan, China) were ultrasonically cleaned in anhydrous ethanol for 5 min and then rinsed with deionized water. Subsequently, the two kinds of Cu foils were dried at 60 °C for 1 h in a vacuum. The targets were positioned at an angle of 20° ± 3° towards the normal direction of the substrate. The distance between the targets and the substrate was 90 ± 5 mm. The sputtering vacuum chamber was pumped down to a base pressure of less than 8 × 10^−4^ Pa, and then the working pressure was fixed at 1 Pa under an Ar atmosphere with a flow rate of 40 standard cubic centimeters per minute (sccm). Additionally, the pre-sputtering (power: 50 W; time: 600 s) was conducted to eliminate oxidized layers and impurities from the surface of targets.

For the co-sputtering process, the Cu was sputtered on the ED Cu foil substrates using RF magnetron sputtering at powers of 25 W, 50 W, 100 W and 150 W, while Al was sputtered at a constant DC power of 150 W. Furthermore, the Cu target was also applied by DC magnetron sputtering with powers of 60 W and 100 W, respectively, when the Al target was kept at a DC power of 50 W. To fabricate Al-Cu alloy films with varying thicknesses, the total co-sputtering times were divided into 600 s, 1800 s, and 3600 s. Two substrate temperatures (room temperature and 170 °C) were chosen to obtain Al-Cu alloys with different initial morphologies. At this time, the DC power applied to the Al target was fixed at 150 W, and the RF power applied to the Cu target was 50 W, 100 W, and 150 W, respectively. Three different substrates (ED Cu foil, RA Cu foil, and Polytetrafluoroethylene (PTFE)) were deposited simultaneously under identical conditions to investigate their effect on the films. Then, two series of bi-layer precursor alloy films were prepared by varying the order of co-sputtering power under continuous deposition conditions. Eventually, all Al-Cu precursor alloy films were dealloyed in a 0.1 M NaOH aqueous solution at room temperature until no visible bubbles were observed on the surface of the samples. Moreover, all dimensions and values were measured 5 times to ensure reproducibility.

### 2.2. Characterization

The phase composition of Al-Cu alloy precursor films was characterized by an XD-3 diffractometer (Beijing Purkinje General Instrument Co., Ltd., Beijing, China) equipped with Cu Kα radiation. The as-dealloyed samples were analyzed by grazing incidence high-resolution X-ray diffractometer (GIXRD, Rigaku SmartLab, Rigaku Corporation, Akishima, Japan). The microstructures of samples before and after dealloying were investigated by a high-resolution scanning electron microscope (SEM, JSM-7610 F, JEOL, Tokyo, Japan). Chemical compositions and elemental distributions were determined using an energy-dispersive X-ray spectrometer (EDS). Ligament size distribution was measured by using the Image J software (1.53a) for at least 100 measurements.

## 3. Results and Discussion

### 3.1. Effect of Sputtering Parameters on Al-Cu Precursor Thin Films

**Figure 1:**
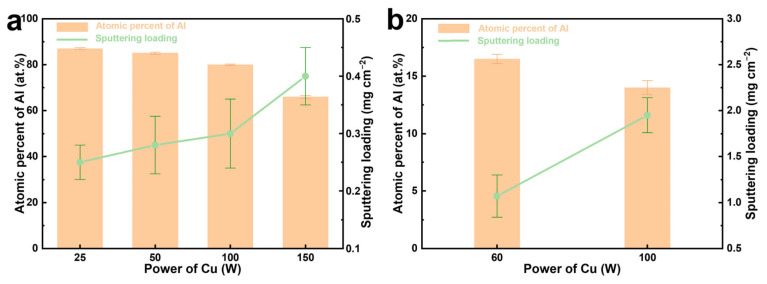
Compositions (Al contents) and sputtering loadings of the Al-Cu films fabricated by adjusting the power of the Cu target while maintaining the power of the Al target at (**a**) DC 150 W and (**b**) DC 50 W. The deposition time parameter was set to 3600 s.

Regarding the deposition using metal targets, it is noted that the DC power supply generally leads to a faster deposition rate than the RF power supply [28]. Moreover, Cu atoms exhibit a higher deposition rate than Al atoms. Consequently, to achieve Al-Cu alloy films with a desired Al atomic ratio, a strategic approach was adopted: employing DC power for the Al target and RF power for the Cu target. Conversely, to obtain precursor films with a preferred Cu atomic ratio, the Cu target was subjected to a DC power supply. Therefore, the Al-Cu alloys with varying compositions can be efficiently fabricated by employing different power combinations for the Al and Cu targets. Figure 1 illustrates the relationship between the applied power, film composition, and sputtering loading. Notably, as the power applied to the Cu target increases, there is a discernible decrease in the Al content within the alloy film. Concurrently, the deposition loading exhibits a significant increase. Macroscopic photographs of these films show a gradient in color, where the Al-Cu films with higher Al contents exhibit a silver-white, while those with increased Cu contents display a yellowish tint (Figure S1).

**Figure 2:**
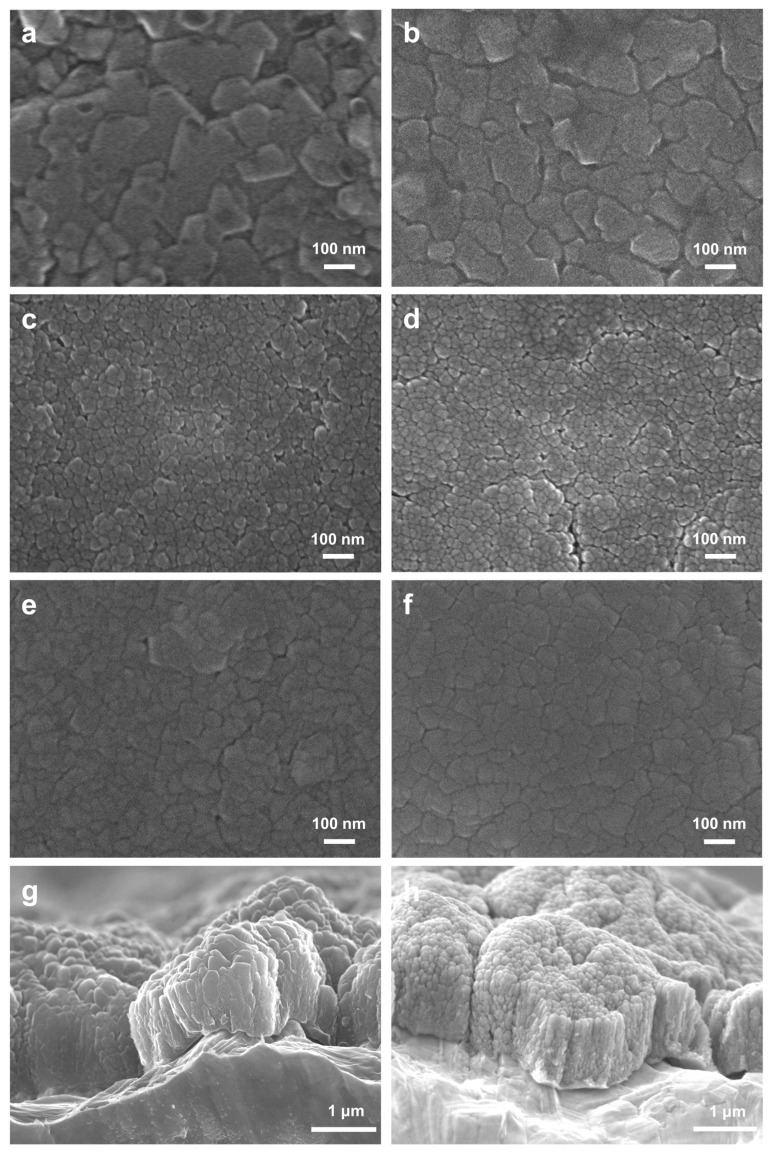
(**a**–**f**) Plan-view SEM images of (**a**) Al_87_Cu_13_, (**b**) Al_85_Cu_15_, (**c**) Al_80_Cu_20_, (**d**) Al_66_Cu_34_, (**e**) Al_17_Cu_83_, and (**f**) Al_12_Cu_88_ on the RA Cu foil substrates. (**g**,**h**) Cross-sectional SEM images of (**g**) Al_80_Cu_20_ and (**h**) Al_66_Cu_34_ on the ED Cu foil substrates. These films were deposited at room temperature and sputtered for 3600 s.

**Figure 3:**
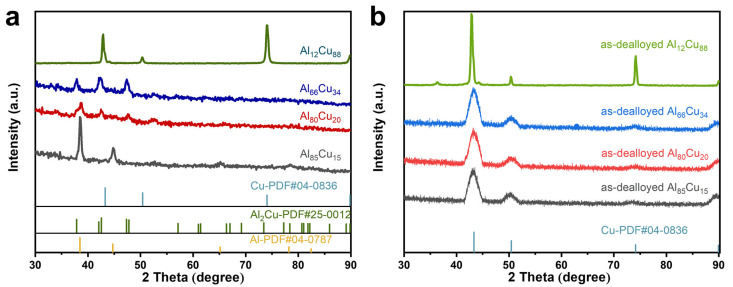
XRD patterns of the Al_85_Cu_15_, Al_80_Cu_20_, Al_66_Cu_34_, and Al_12_Cu_88_ films (**a**) before and (**b**) after dealloying.

Following this observation, compositional analysis was performed on the samples utilizing EDS, which identified six distinct compositions of Al-Cu alloy precursor films: Al_87_Cu_13_, Al_85_Cu_15_, Al_80_Cu_20_, Al_66_Cu_34_, Al_17_Cu_83_, and Al_12_Cu_88_ (Figure S2). As shown in Figure 2a–h, the Al-Cu alloy films are composed of irregularly shaped columnar grains. Notably, there is a conspicuous decrease in grain size, which varies from 139.5 nm to 28.3 nm with increasing Cu content until reaching 34 at.% (Figure S3). This phenomenon is attributed to the gradual transition of the alloy from a single-phase solid solution (α-Al) to an Al_2_Cu intermetallic compound as the Cu content increases (Figure 3a). It is worth noting that the resulting grain size is a micro- or nanostructural parameter that has an important role in the attained properties, e.g., corrosion resistance and mechanical behavior. It is reported that a finer grain size provides an improvement on the mechanical strength, while a coarser one can significantly affect the corrosion behavior [29,30,31,32]. The peaks at 2θ = 38.472°, 44.738°, 65.133°, and 78.227° are well indexed to the face-centered cubic (fcc) α-Al phase (PDF # 04-0787) in the Al_85_Cu_15_ film. Peaks at 37.866°, 42.07°, 42.59°, 47.331°, and 47.807° correspond to the Al_2_Cu phase (PDF # 25-0012) in the Al_66_Cu_34_ film. Noticeably, the Al_80_Cu_20_ film is composed of two phases: α-Al and Al_2_Cu. In addition, the peaks located at 43.297°, 50.433°, 74.13°, and 89.931° indicate that only the Cu phase (PDF # 04-0836) can be identified in the Al_12_Cu_88_ film. However, when the Cu atom ratio reaches 83 at.% or 88 at.%, the phase composition shifts to a single solid solution, leading to an increase of the grain size. The cross-sectional SEM results (Figure 2g,h) reveal that the columnar grains are densely arranged together to form large island-like structures.

Subsequently, the relationship between the thickness of Al-Cu precursor films and sputtering time was systematically investigated. As shown in Figure S4, the Al-Cu precursor films with various compositions (Al_85_Cu_15_, Al_80_Cu_20_, Al_66_Cu_34_) were deposited for the duration of 600 s, 1800 s, and 3600 s, respectively. For all the Al-Cu films, the thickness shows an approximately linear correlation with the sputtering time. It is noteworthy that the thickness of the film not only impacts the stability of the nanoporous films obtained through subsequent dealloying but also influences the morphology and ligament size of the resulting nanoporous structures [23]. Hence, precise control of film thickness emerges as a crucial strategy for modulating nanoporous structures.

In order to explore the influence of various substrates on both film composition and morphology, the Al_12_Cu_88_ alloys were simultaneously deposited on three distinct substrates (ED Cu foil, polished RA Cu foil, PTFE) at the same power settings, resulting in the production of three sample films. Morphological characterizations of the samples reveal variations in the morphology and roughness of the substrates, leading to differences in the microstructures of the obtained films (Figure S5). Obviously, the microstructure of the sputtered films is inherently influenced by the shape and structure of the substrate, leading to an inheritance of the substrate surface pattern [33]. This phenomenon can be attributed to varying degrees of shadowing effects during deposition, where sputtered atoms tend to nucleate more readily at the high points of a rough surface compared to the valleys [34]. The accumulation of adsorbed sputtered atoms at these high points facilitates their diffusion into the adjacent valleys [35], ultimately resulting in the formation of a relatively dense film. Comparing the RA and ED Cu foils (Figure S5a,e) to PTFE (Figure S5c), there is a noticeable increase in the presence of white aggregates in the latter. It is hypothesized that due to the fibrous nature of the PTFE substrate, a significant number of sputtered atoms experience more collisions as they infiltrate the fiber voids on the substrate surface during deposition, thereby enhancing the likelihood of agglomerate nucleation. While white aggregates are also observed in the Al_12_Cu_88_ film supported on the RA and ED Cu foils, they appear in minimal quantities, which can be attributed to the relatively flat surfaces in these cases. In accordance with the structural zone model proposed by the Thornton diagram [36,37], the formation of Al_12_Cu_88_ at room temperature falls within zone 1 (T/Tm < 0.3). In this zone, the film comprises a dense array of elongated, small-diameter fiber crystals (Figure S6), with the shadowing effect prevailing [38]. Despite considerable variations in the microscopic morphology of the films formed on different substrates, the EDS results (Figure S7) indicate minimal disparity in chemical compositions across the three samples.

### 3.2. Effect of Film Composition on the Microstructures of np-Cu

**Figure 4:**
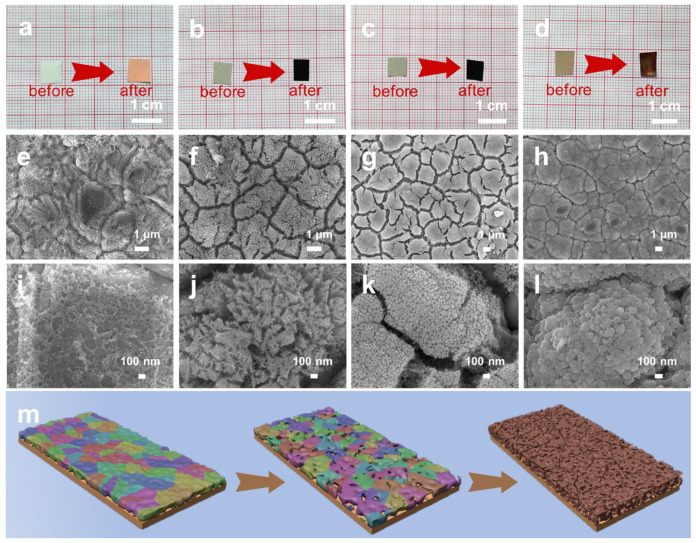
(**a**–**d**) Optical images of (**a**) Al_85_Cu_15_, (**b**) Al_80_Cu_20_, (**c**) Al_66_Cu_34_, and (**d**) Al_12_Cu_88_ before and after dealloying. (**e**–**l**) SEM images of the np-Cu films dealloyed from (**e**,**i**) Al_85_Cu_15_, (**f**,**j**) Al_80_Cu_20_, (**g**,**k**) Al_66_Cu_34_, and (**h**,**l**) Al_12_Cu_88_. These films were deposited onto ED Cu foils at room temperature with a sputtering time of 3600 s. (**m**) Schematic illustrations showing the microstructure evolution of Al_66_Cu_34_ during dealloying.

Then we selected the Al_85_Cu_15_, Al_80_Cu_20_, Al_66_Cu_34_, and Al_12_Cu_88_ precursor films to investigate the influence of precursor composition on the microstructures of np-Cu. The XRD patterns of four as-dealloyed precursor films reveal the single face-centered cubic (fcc) Cu phase (PDF # 04-0836) (Figure 3b), illustrating that most of the Al was removed from the precursor films, which is further confirmed by the EDS results (Figure S8). Moreover, the residual Al content is less than 3 at.% in the four np-Cu films. Figure 4a–d shows the optical images of four thin films before and after dealloying. Clearly, the Al_80_Cu_20_ and Al_66_Cu_34_ precursor films become dark after dealloying, implying the formation of a nanoporous structure, which can efficiently enhance light absorption by providing numerous nanoscale pores. In contrast, the as-dealloyed Al_85_Cu_15_ and Al_12_Cu_88_ films exhibit orange-red and reddish-brown colors, respectively, which can be attributed to the absence of internal ligament-channel penetration structure of the two as-dealloyed films (Figure 4e,h,i,l). The SEM images in Figure 4e,i further reveal that the np-Cu dealloyed from Al_85_Cu_15_ was severely detached from the substrate due to volume shrinking during dealloying [39], with only a small amount of ligaments remaining to cover the surface. With increasing initial Cu content to 20 at.% (Figure 4f,j), the detachment of np-Cu is significantly improved, and the np-Cu evenly covers the surface of the substrate. In particular, the dealloying along the columnar grain boundaries occurred in addition to within the grains due to the limited reorganizational capacity of the Cu atoms [40], forming a multiscale channel structure (gaps formed by dealloying columnar grain boundaries and nanopores formed by dealloying columnar grains). The ligament size within the columnar grains is around 13.3 nm (Figure S9a). However, for the np-Cu sample dealloyed from Al_66_Cu_34_ (Figure 4g,k), the gaps formed by dealloying columnar grain boundaries at the top surface are not visible, and the nanoporous structure is isotropic with a ligament size of about 19 nm (Figure S9b) owing to more reorganization during dealloying. As the np-Cu dealloyed from Al_12_Cu_88_ (Figure 4h,l), the interpenetrating ligament/pore structure disappears, and a nanoparticle stacking structure emerges due to the predominance of Cu atoms. This phenomenon can be elucidated by the high Cu content, which results in the space formed after dealloying Al being too small to facilitate the diffusion and recombination of Cu atoms to form a nanoporous skeleton. Based on the above results, it can be seen that the different phase compositions of initial precursor films with similar structures elucidate the substantial differences in nanoporous morphology after dealloying.

Figure 4m vividly illustrates the formation process of the np-Cu films during the dealloying of the Al-Cu precursors. The initial Al-Cu precursor film exhibits a random grain orientation. Upon immersion in the alkaline solution, the dealloying first occurred at the grain boundaries due to their particularly pronounced chemical activity, and then the corrosive solution penetrated downward and caused lateral etching [41], forming small holes on the surface. As dealloying proceeded, a large number of Al atoms were selectively leached out, while the released Cu atoms diffused along the alloy/solution interface and re-organized to form the bi-continuous nanoporous structure [41]. Additionally, after dealloying, the film thickness decreased, accompanied by volume contraction and the development of microcracks within the np-Cu film.

### 3.3. Effect of Substrate Temperature on the Microstructure of np-Cu

Based on the structural zone model, it can be seen that the substrate temperature determines the physical process of atomic motion, which has a significant effect on the microstructure of the Al-Cu precursor films. The physical processes of atomic motion encompass the shadow effect, surface diffusion, and bulk diffusion. Depending on the dominant physical processes, the film structure transitions from fibrous crystals to columnar crystals and finally to equiaxed crystals. Simultaneously, the crystal diameter increases, and the density of the film rises with increasing substrate temperature [42,43]. Therefore, microstructure regulation of np-Cu can be achieved by adjusting the substrate temperature. Herein, taking the Al_66_Cu_34_ precursor film as an example, two distinct substrate temperatures (room temperature and 170 °C) were selected during sputtering. For these substrate temperatures, since the ratio between substrate temperature and melting point (T/T_m_) is greater than 0.3 and less than 0.5, which corresponds to zone 2 of the Thornton structure diagram, the microstructure of precursor films exhibits parallel columnar crystallites arranged in a circular pattern and oriented perpendicular to the substrate. As shown in Figure S10a,b, when the sputtering time is 1800 s, the precursor film deposited at room temperature consists of irregular columnar crystallites with a triangular top, while larger grain size and regular columnar structures with a hemispherical top are formed in the precursor film owing to higher substrate temperature (170 °C) (Figure S10c,d). With decreasing sputtering time to 600 s (Figure S10e–h), the columnar structure of precursor films becomes unapparent due to its insufficient thickness caused by shorter deposition time.

**Figure 5:**
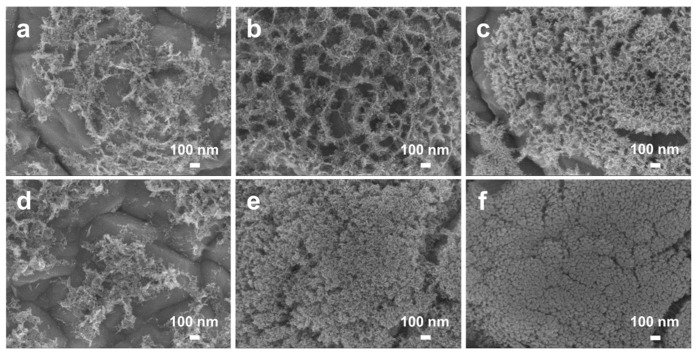
Plan-view SEM images of the np-Cu films dealloyed from (**a**,**d**) Al_85_Cu_15_, (**b**,**e**) Al_80_Cu_20_, and (**c**,**f**) Al_66_Cu_34_. These thin films were deposited onto the ED Cu foils at (**a**–**c**) at room temperature and (**d**–**f**) 170 °C for 1800 s.

Subsequently, three precursor films (Al_85_Cu_15_, Al_80_Cu_20_, Al_66_Cu_34_) sputtered using various substrate temperatures and sputtering times were dealloyed to obtain np-Cu samples (Figure 5). For the np-Cu dealloyed from Al_85_Cu_15_ sputtered at room temperature (Figure 5a), the detachment of np-Cu from the substrate occurs and only a small number of np-Cu ligaments cover the surface of the substrate. When the substrate temperature was heated to 170 °C, the remaining np-Cu ligaments tended to aggregate into clusters (Figure 5d). For the np-Cu film dealloyed from Al_80_Cu_20_ sputtered at room temperature (Figure 5b), a multiscale honeycomb structure consisting of fine ligaments/channels was observed after dealloying, with an average ligament size of 11.4 nm (Figure S11a). In comparison, for the Al_80_Cu_20_ film prepared at 170 °C, the obtained np-Cu film shows a bi-continuous ligament-channel structure (Figure 5e) with a ligament size of 12.1 nm (Figure S11c). For the np-Cu dealloyed from Al_66_Cu_34_ (sputtered at room temperature), the honeycomb structure becomes denser (average ligament size: 13.4 nm, Figure 5c and Figure S11b), while the ligaments of the sample prepared from Al_66_Cu_34_ sputtered at 170 °C (Figure 5f) were obviously coarsened (average ligament size: 18.2 nm, Figure S11d). Clearly, as the substrate temperature increases, the morphology of the formed np-Cu changes, accompanied by an increase in the ligament size.

### 3.4. Bi-Layer np-Cu with a Hierarchical Structure

Hierarchically porous materials used as catalysts exhibit exceptional attributes such as high activity, rapid diffusion rates, and superior stability compared to their bulk counterparts [10]. So far, synthesis strategies for hierarchically porous nanostructures have been hampered by the drawbacks of involving multiple steps, extended reaction durations, and high preparation temperatures [44]. Magnetron sputtering has been employed to produce numerous hierarchical nanoporous metals through one-step direct dealloying. Following the aforementioned experiments, we chose two film compositions, Al_66_Cu_34_ and Al_80_Cu_20_, as precursors for creating bi-layer nanoporous films. By manipulating the deposition sequence of the two compositions, we obtained two precursor films: Al_66_Cu_34_-on-Al_80_Cu_20_ and Al_80_Cu_20_-on-Al_66_Cu_34_, which enabled us to prepare bi-layer nanoporous films and investigate the effect of the deposition sequence on the delamination of the porous structure.

**Figure 6:**
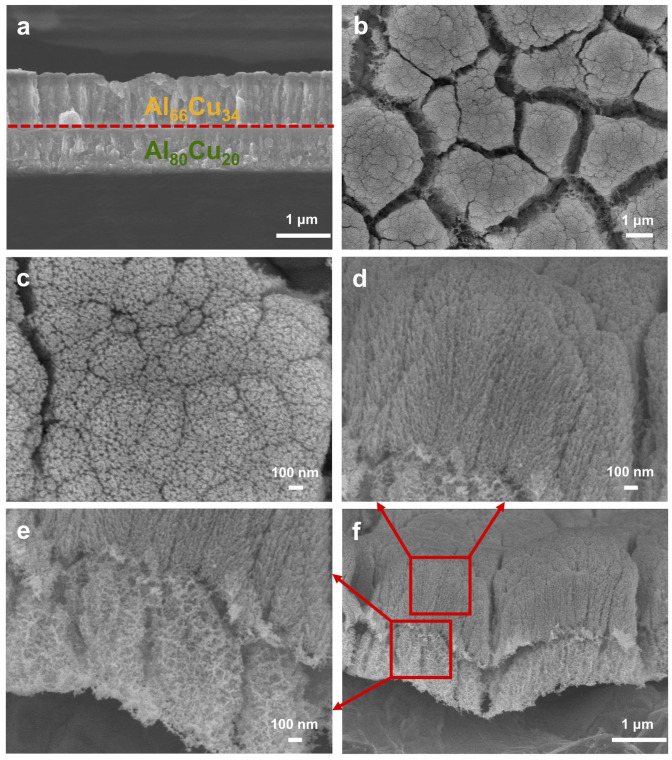
(**a**) Cross-sectional SEM image of the Al_66_Cu_34_-on-Al_80_Cu_20_ film. (**b**,**c**) Plan-view and (**d**–**f**) cross-sectional SEM images of the np-Cu film dealloyed from Al_66_Cu_34_-on-Al_80_Cu_20_. Each layer was sputtered at room temperature for 3600 s.

Figure 6 illustrates the plan-view and cross-sectional SEM images of the Al_66_Cu_34_-on-Al_80_Cu_20_ before and after dealloying. It can be seen that the bi-layer precursor film consists of two Al-Cu alloys with different compositions: the top Al_66_Cu_34_ layer and the bottom Al_80_Cu_20_ layer with a distinct interface between the two layers (Figure 6a). Meanwhile, similar dense columnar crystals can be observed in these two layers. After dealloying, for the top np-Cu layer dealloyed from Al_66_Cu_34_, the surface exhibits grain-like morphology with an interconnected network of channels (Figure 6b). Additionally, high-magnification plan-view SEM images (Figure 6c and Figure S12) show that many irregular boundary cracks can be clearly observed within each individual grain-like nanoporous island, indicating that the columnar crystal structure is retained due to the corrosion of the columnar crystal boundaries of the precursor film during dealloying [45]. At the same time, an isotropic, bi-continuous ligament-channel structure forms within each single columnar grain. The cross-section SEM images (Figure 6d–f) reveal two types of nanoporous structure with the more distinguished interface between the top and bottom layers. The bottom np-Cu layer also preserves the pristine columnar grain structure but is not obvious. Furthermore, the larger columnar grain size and ligament/pores are formed (Figure 6e), compared with the top np-Cu layer.

**Figure 7:**
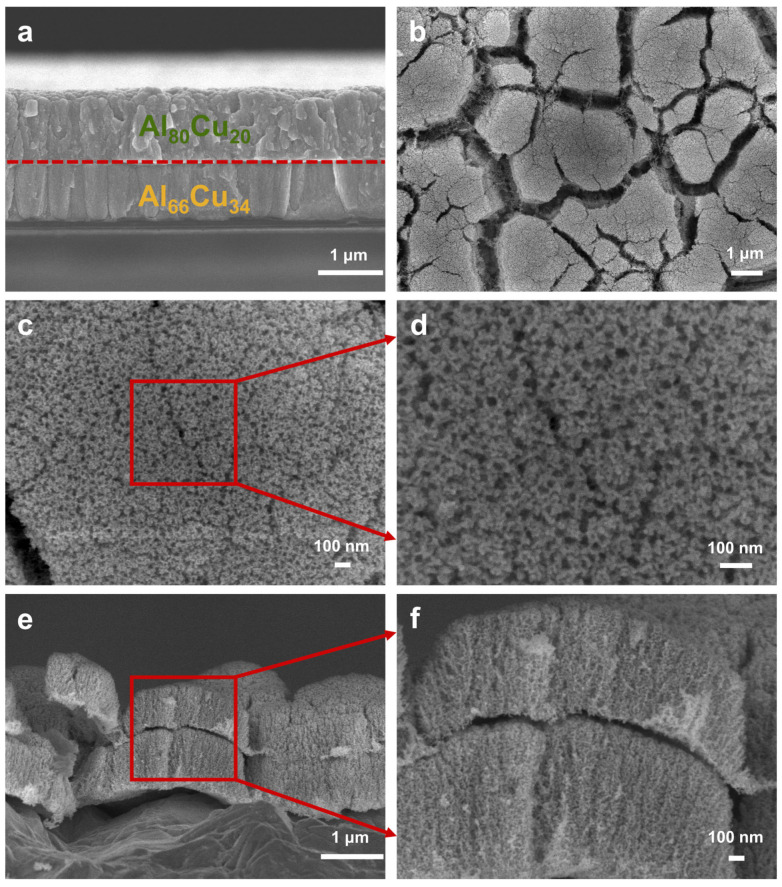
(**a**) Cross-sectional SEM image of the Al_80_Cu_20_-on-Al_66_Cu_34_ film. (**b**–**d**) Plan-view and (**e**,**f**) cross-sectional SEM images of the np-Cu film dealloyed from Al_80_Cu_20_-on-Al_66_Cu_34_. Each layer was sputtered at room temperature for 3600 s.

For the Al_80_Cu_20_-on-Al_66_Cu_34_ film, the top and bottom layers both consist of columnar crystals (Figure 7a). The crystal structure of Al_66_Cu_34_ is more pronounced compared to the same composition in Figure 6a. After dealloying, the plan-view and cross-section SEM images (Figure 7b–f) illustrate that different from the scenario of Al_66_Cu_34_-on-Al_80_Cu_20_, the bi-layer np-Cu film displays an isotropic interconnected nanoporous structure with similar characteristics in both top and bottom layers, and the columnar crystal structure is difficult to identify. In addition, the ligament size of the top np-Cu layer dealloyed from Al_80_Cu_20_ is marginally smaller than that of the bottom layer dealloyed from Al_66_Cu_34_ (Figure 7f and Figure S13). Obviously, altering the sequence of film deposition results in the growth of films on distinct substrates, consequently leading to variations in the microstructure of the precursor film. Correspondingly, the precursor films with the same composition grown on different substrates lead to the formation of different nanostructures after dealloying, as observed in our experiments.

## 4. Conclusions

In summary, by providing a detailed understanding of the relationships between sputtering parameters and precursor design, Al-Cu precursor films with different compositions and morphologies were fabricated by the magnetron co-sputtering technique. One-step dealloying strategy was subsequently utilized to fabricate isotropic np-Cu films. The results reveal that the sputtering power and substrate temperature have an obvious effect on the formation and microstructure of the Al-Cu films. Indeed, the ligament size of np-Cu could be adjusted within the range of 11.4 to 19.0 nm. The precursor structure and the resulting np-Cu morphology are tuned with the increase in substrate temperature, leading to an increase in ligament size and structural compactness. Moreover, bi-layer Al-Cu precursor films were prepared through altering the deposition sequence of monolayer films with varying compositions and were further dealloyed to create bi-layer np-Cu films with distinct interfaces and porous structures. The present work provides valuable information on the design, fabrication, and structural regulation of nanoporous metal films. Future studies could further optimize the nanoporous metal synthesis process based on our findings and investigate the properties of these materials in different application conditions.

## Data Availability

The original contributions presented in this study are included in the article/Supplementary Material. Further inquiries can be directed to the corresponding authors.

## Supplementary Materials

The following supporting information can be downloaded at: https://www.mdpi.com/article/10.3390/ma17235705/s1, Figure S1. The optical photographs of (a) Al_87_Cu_13_, (b) Al_85_Cu_15_, (c) Al_80_Cu_20_, (d) Al_66_Cu_34_, (e) Al_17_Cu_83_, and (f) Al_12_Cu_88_. Figure S2. The EDS results of (a) Al_87_Cu_13_, (b) Al_85_Cu_15_, (c) Al_80_Cu_20_, (d) Al_66_Cu_34_, (e) Al_17_Cu_83_, and (f) Al_12_Cu_88_. Figure S3. Grain size of the sputtered Al-Cu alloy films. Figure S4. (a,d,g) The film thickness of Al_85_Cu_15_ sputtered for 3600 s, 1800 s, and 600 s, respectively. (b,e,h) The film thickness of Al_80_Cu_20_ sputtered for 3600 s, 1800 s, and 600 s, respectively. (c,f,i) The film thickness of Al_66_Cu_34_ sputtered for 3600 s, 1800 s, and 600 s, respectively. These films were deposited onto the RA Cu foils at room temperature. Figure S5. Plan-view SEM images of Al_12_Cu_88_ (a,b) on the RA Cu foil substrate, (c,d) PTFE, and (e,f) ED Cu foil substrate. These films were deposited at room temperature with a sputtering time of 3600 s. Figure S6. Cross-sectional SEM images of the Al_12_Cu_88_ on ED Cu foil substrate. Figure S7. The EDS results of the Al_12_Cu_88_ films sputtered on the (a) RA Cu foil substrate, (b) PTFE, and (c) ED Cu foil substrate. Figure S8. The EDS results of the np-Cu films dealloyed from the (a) Al_85_Cu_15_, (b) Al_80_Cu_20_, (c) Al_66_Cu_34_ and (d) Al_12_Cu_88_ precursors. Figure S9. Ligament size distribution of the np-Cu films dealloyed from (a) Al_80_Cu_20_ and (b) Al_66_Cu_34_. These films were deposited at room temperature for 3600 s. Figure S10. Plan-view SEM images of Al_66_Cu_34_ sputtered for (a,c) 1800 s and (e,g) 600 s. Cross-sectional SEM images of Al_66_Cu_34_ sputtered for (b,d) 1800 s and (f,h) 600 s. These thin films were deposited (a,b,e,f) at room temperature, (c,d,g,h) at 170 °C. Figure S11. Ligament size distribution of the np-Cu films dealloyed from (a,c) Al_80_Cu_20_ and (b,d) Al_66_Cu_34_. These films were deposited at (a,b) room temperature and (c,d) 170 °C for 1800 s. Figure S12. The high-magnification plan-view SEM image of the bilayer np-Cu film dealloyed from the Al_66_Cu_34_-on-Al_80_Cu_20_ precursor. Figure S13. Ligament size distribution of (a) upper and (b) lower layers of the np-Cu film dealloyed from Al_80_Cu_20_-on-Al_66_Cu_34_.
