# Supplementary material for: Synthesis and Structural Modulation of Nanoporous Copper Films by Magnetron Sputtering and One-Step Dealloying
Source: Materials (Basel). 2024 Nov 21;17(23):5705. doi: 10.3390/ma17235705 (PMC11641967; doi:10.3390/ma17235705)
Supplement: Supplementary file 1 [file materials-17-05705-s001.zip › materials-3317263-supplementary.pdf]

Supporting Information:

Synthesis and structural modulation of nanoporous copper films by magnetron sputtering and one-step dealloying

Jinglei Li, Bin Yu, Yunfei Ran, Yalong Liu, Xiangyu Fei, Jiameng Sun, Fuquan Tan, Guanhua Cheng, Ying Zhang *, Jingyu Qin, Zhonghua Zhang *

Key Laboratory for Liquid-Solid Structural Evolution and Processing of Materials (Ministry of Education), School of Materials Science and Engineering, Shandong University, Jingshi Road 17923, Jinan 250061, P.R. China

*Corresponding author. Email: zh_zhang@sdu.edu.cn (Z. Zhang); ying_zhang7@hotmail.com (Y. Zhang)

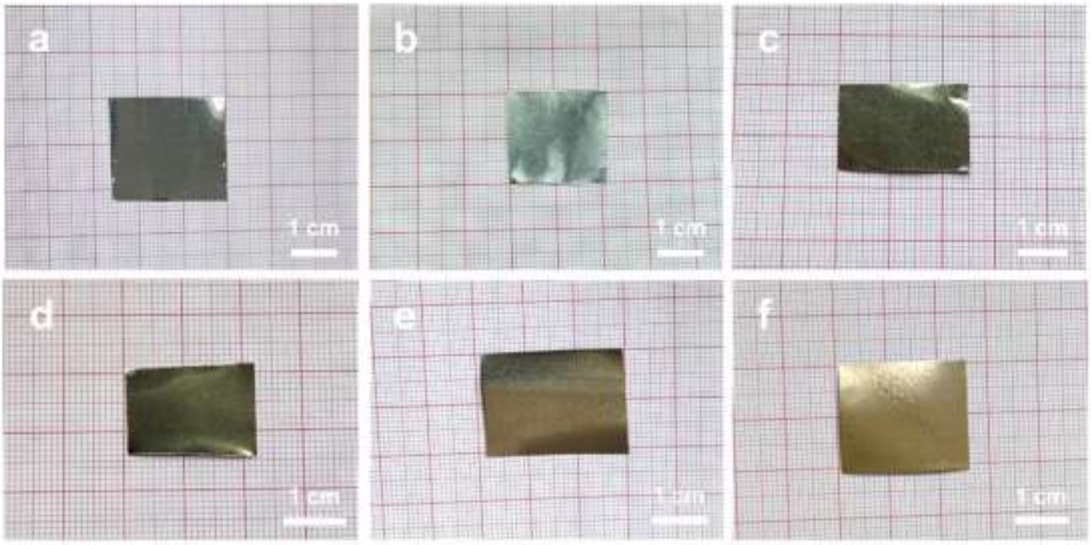

Figure S1. The optical photographs of (a) $Al_{87}Cu_{13}$, (b) $Al_{85}Cu_{15}$, (c) $Al_{80}Cu_{20}$, (d) $Al_{66}Cu_{34}$, (e) $Al_{17}Cu_{83}$, and (f) $Al_{12}Cu_{88}$.

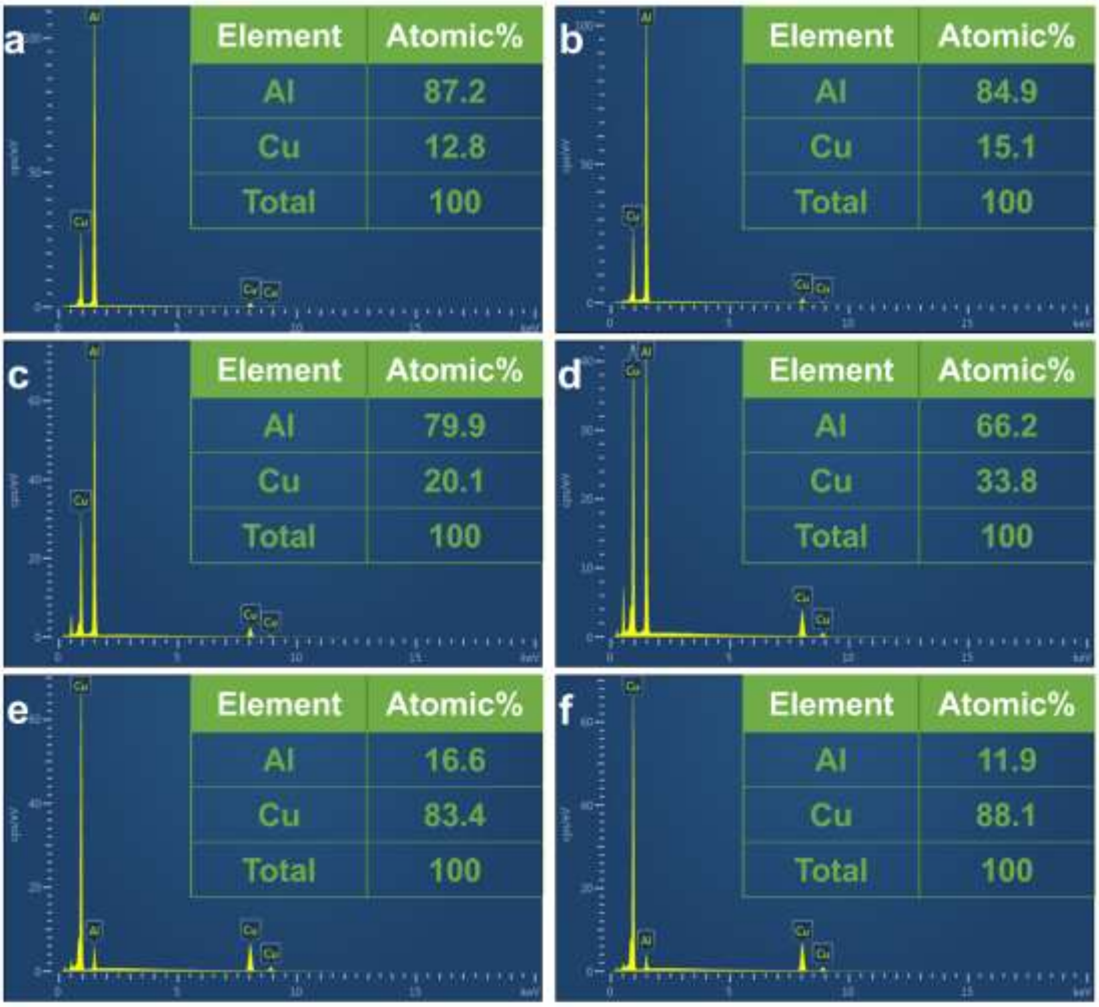

Figure S2. The EDS results of (a) $Al_{87}Cu_{13}$, (b) $Al_{85}Cu_{15}$, (c) $Al_{80}Cu_{20}$, (d) $Al_{66}Cu_{34}$, (e) $Al_{17}Cu_{83}$, and (f) $Al_{12}Cu_{88}$.

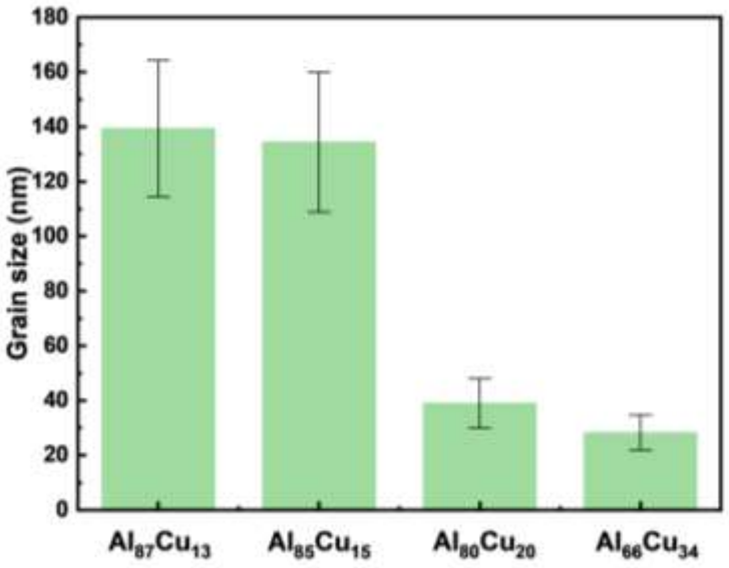

Figure S3. Grain size of the sputtered Al-Cu alloy films.

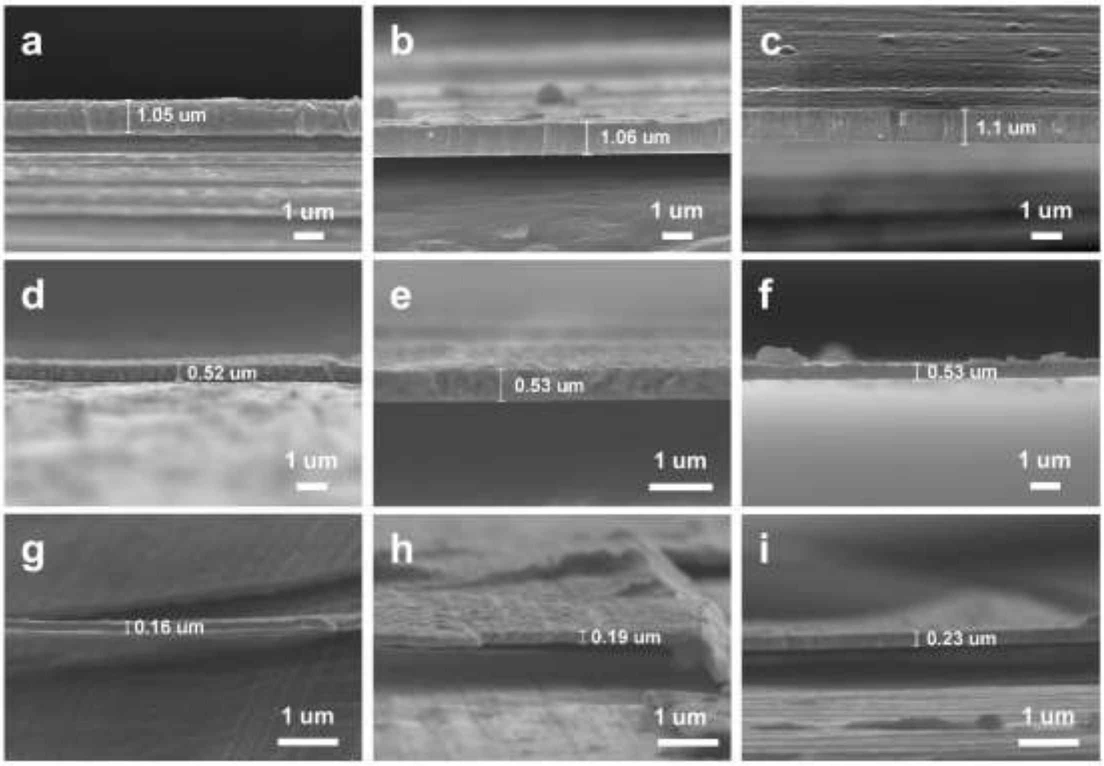

Figure S4. (a,d,g) The film thickness of $Al_{85}Cu_{15}$ sputtered for 3600 s, 1800 s, and 600 s, respectively. (b,e,h) The film thickness of $Al_{80}Cu_{20}$ sputtered for 3600 s, 1800 s, and 600 s, respectively. (c,f,i) The film thickness of $Al_{66}Cu_{34}$ sputtered for 3600 s, 1800 s, and 600 s, respectively. These films were deposited onto the RA Cu foils at room temperature.

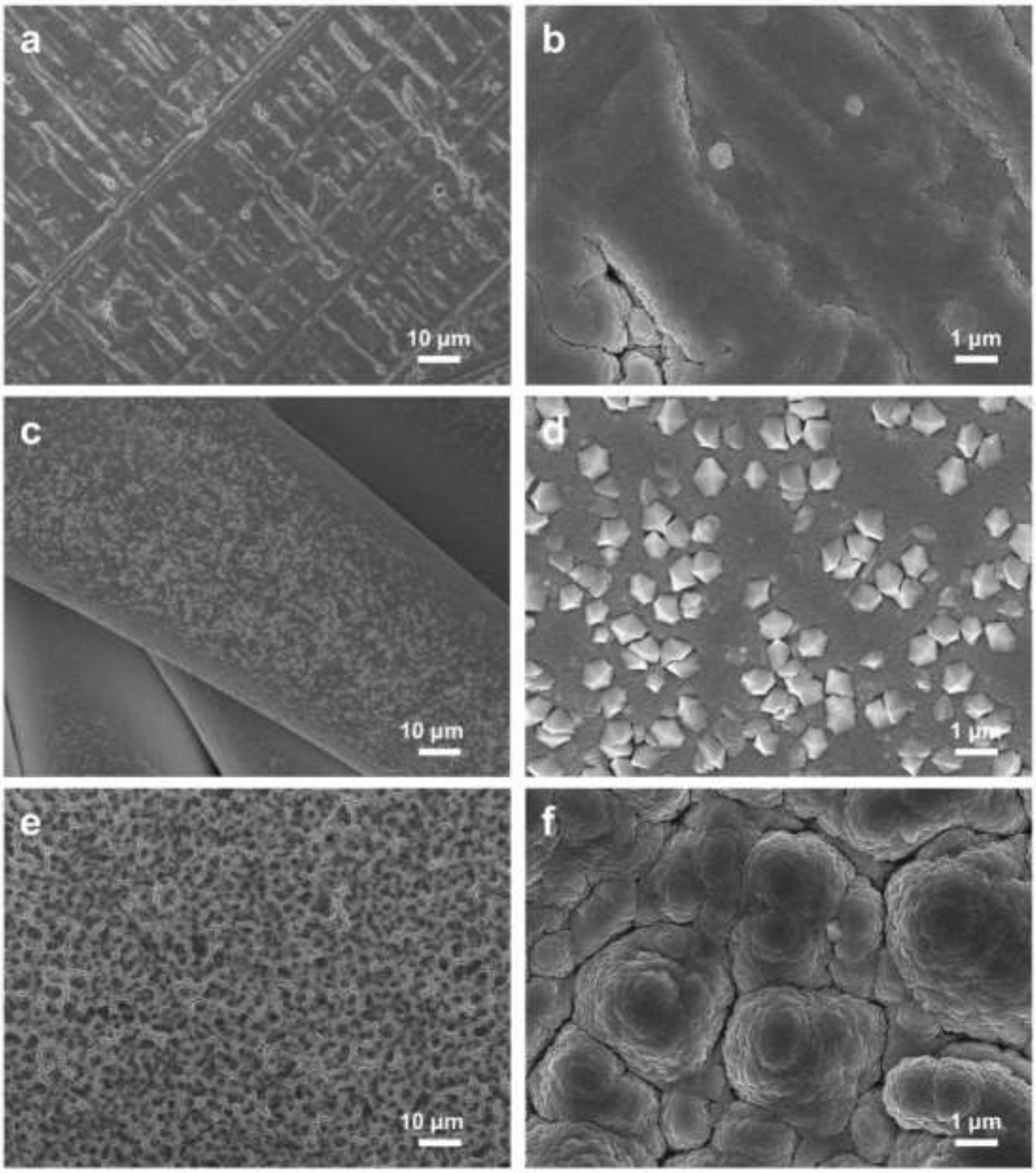

Figure S5. Plan-view SEM images of $Al_{12}Cu_{88}$ (a,b) on the RA Cu foil substrate, (c,d) PTFE, (e,f) and ED Cu foil substrate. These films were deposited at room temperature with a sputtering time of 3600 s.

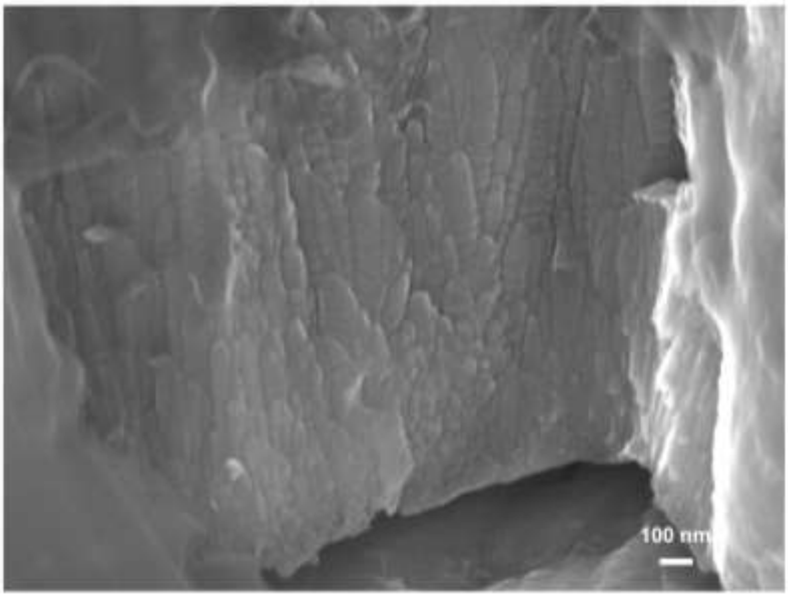

Figure S6. Cross-sectional SEM images of the $Al_{12}Cu_{88}$ on ED Cu foil substrate.

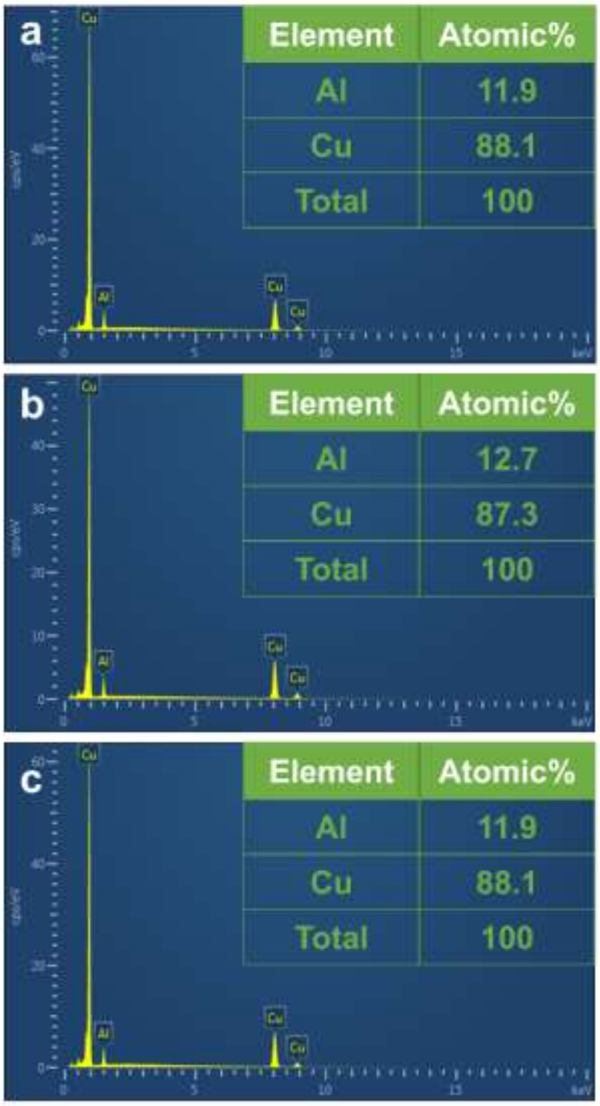

Figure S7. The EDS results of the $Al_{12}Cu_{88}$ films sputtered on the (a) RA Cu foil substrate, (b) PTFE and (c) ED Cu foil substrate.

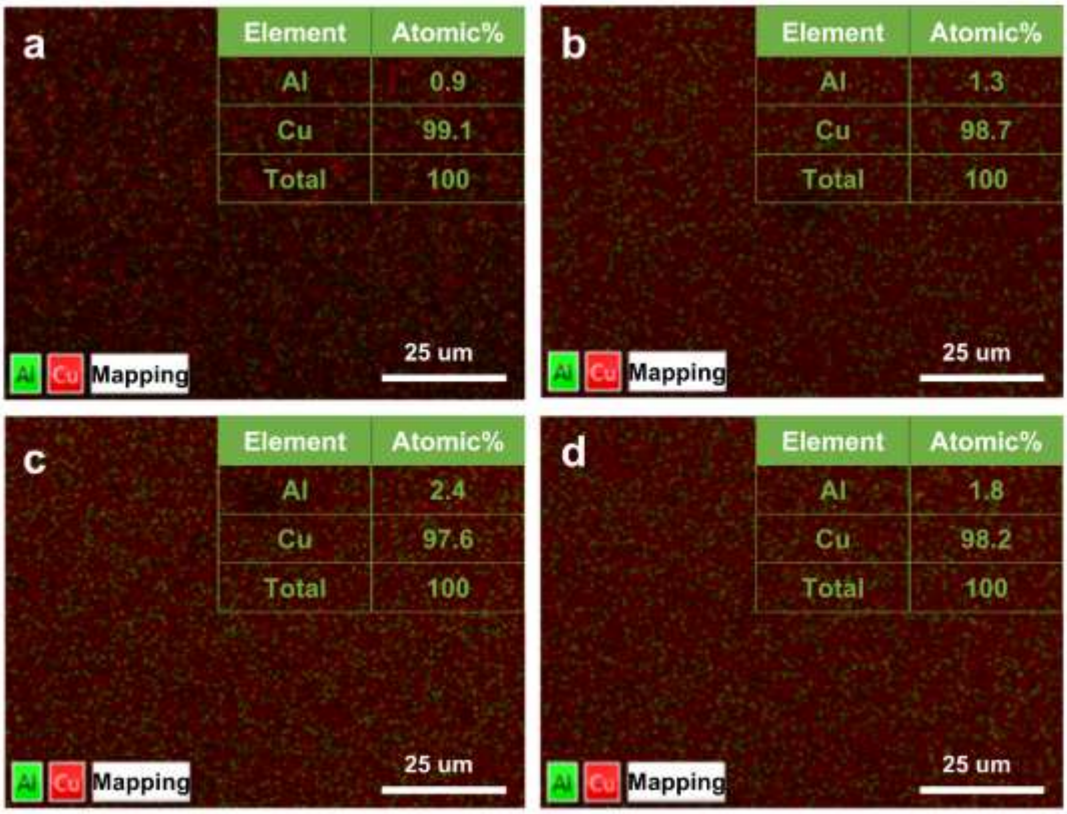

Figure S8. The EDS results of the np-Cu films dealloyed from the (a) $Al_{85}Cu_{15}$, (b) $Al_{80}Cu_{20}$, (c) $Al_{66}Cu_{34}$ and (d) $Al_{12}Cu_{88}$ precursors.

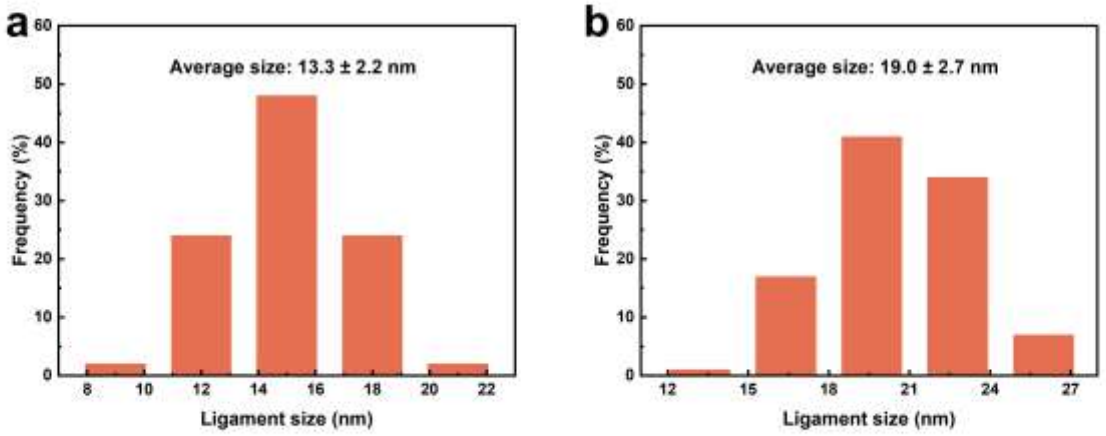

Figure S9. Ligament size distribution of the np-Cu films dealloyed from (a) $Al_{80}Cu_{20}$ and(b) $Al_{66}Cu_{34}$. These films were deposited at room temperature for 3600 s.

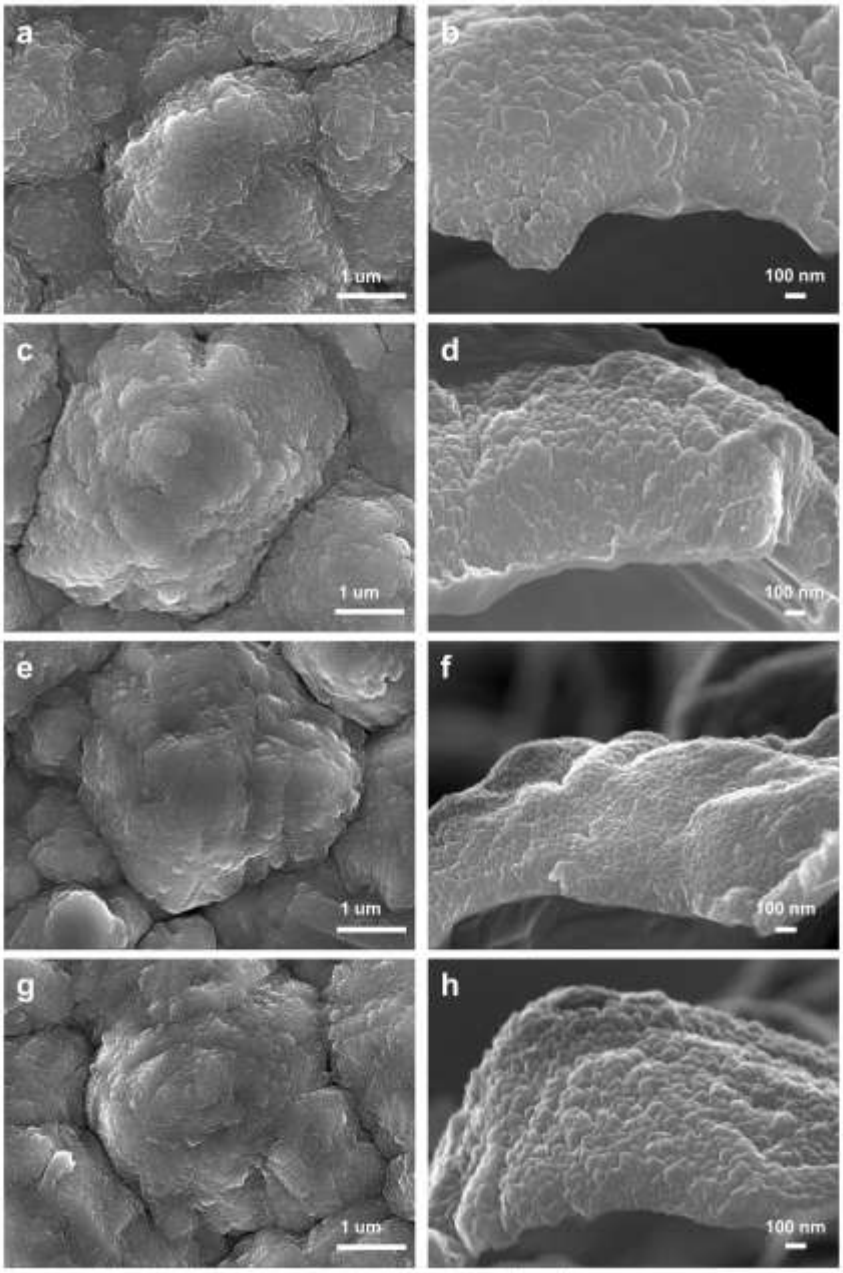

Figure S10. Plan-view SEM images of $Al_{66}Cu_{34}$ sputtered for (a,c) 1800 s and (e,g) 600 s. Cross-sectional SEM images of $Al_{66}Cu_{34}$ sputtered for (b,d) 1800 s and (f,h) 600 s. These thin films were deposited (a,b,e,f) at room temperature, (c,d,g,h) at 170 °C.

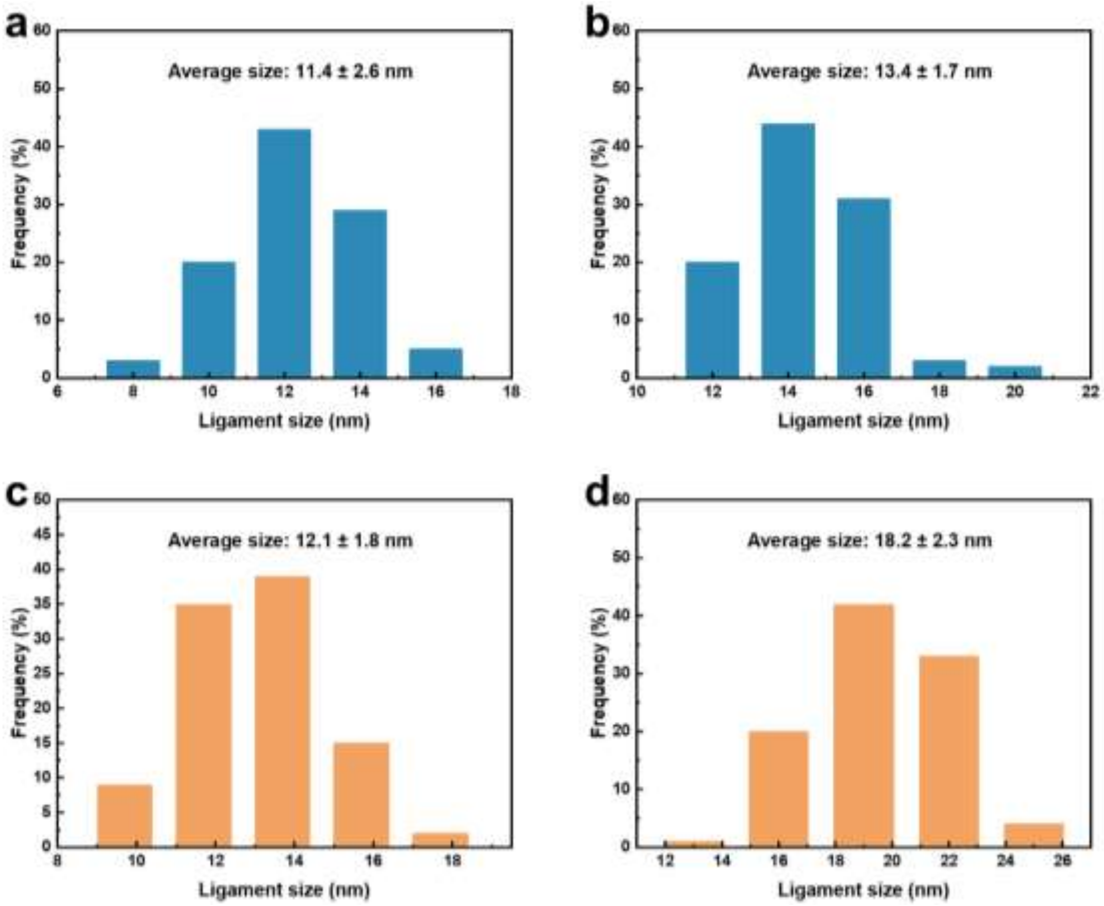

Figure S11. Ligament size distribution of the np-Cu films dealloyed from (a,c) $Al_{80}Cu_{20}$ and (b,d) $Al_{66}Cu_{34}$. These films were deposited at (a,b) room temperature and (c,d) 170 °C for 1800 s.

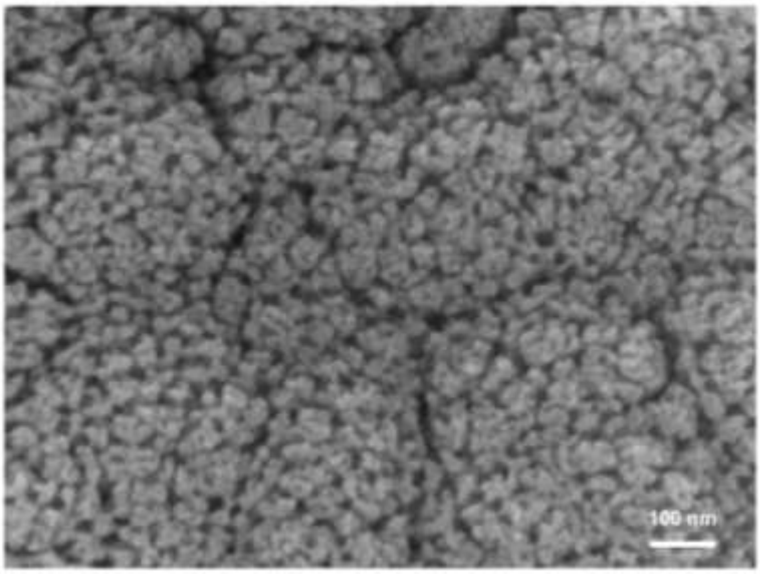

Figure S12. The high-magnification plan-view SEM image of the bilayer np-Cu film dealloyed from the $Al_{66}Cu_{34}$-on-$Al_{80}Cu_{20}$ precursor.

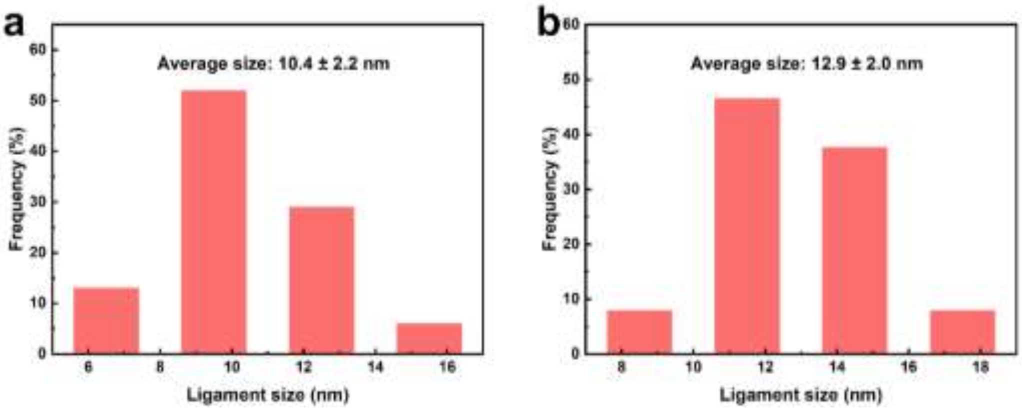

Figure S13. Ligament size distribution of (a) upper and (b) lower layers of the np-Cu film dealloyed from $Al_{80}Cu_{20}$-on-$Al_{66}Cu_{34}$.
